# Dopamine D1-like receptors in the dorsomedial prefrontal cortex regulate contextual fear conditioning

**DOI:** 10.1007/s00213-018-5162-7

**Published:** 2019-01-17

**Authors:** Christine Stubbendorff, Ed Hale, Helen J. Cassaday, Tobias Bast, Carl W. Stevenson

**Affiliations:** 1 School of Biosciences, University of Nottingham, Sutton Bonington Campus, Loughborough, LE12 5RD UK; 2 School of Psychology@Nottingham, University of Nottingham, University Park, Nottingham, NG7 2RD UK; 3 School of Neuroscience@Nottingham, University of Nottingham, University Park, Nottingham, NG7 2RD UK

**Keywords:** Anterior cingulate cortex, Contextual fear, Dopamine, D1 receptor, Nucleus accumbens, Open field, Prefrontal cortex, SCH23390, Ventral hippocampus

## Abstract

**Rationale:**

Dopamine D1 receptor (D1R) signalling is involved in contextual fear conditioning. The D1R antagonist SCH23390 impairs the acquisition of contextual fear when administered systemically or infused locally into the dorsal hippocampus or basolateral amygdala.

**Objectives:**

We determined if state dependency may account for the impairment in contextual fear conditioning caused by systemic SCH23390 administration. We also examined if the dorsomedial prefrontal cortex (dmPFC), nucleus accumbens (NAc), and ventral hippocampus (VH) are involved in mediating the effect of systemic SCH23390 treatment on contextual fear conditioning.

**Methods:**

In experiment 1, SCH23390 (0.1 mg/kg) or vehicle was given before contextual fear conditioning and/or retrieval. In experiment 2, SCH23390 (2.5 μg/0.5 uL) or vehicle was infused locally into dmPFC, NAc, or VH before contextual fear conditioning, and retrieval was tested drug-free. Freezing was quantified as a measure of contextual fear.

**Results:**

In experiment 1, SCH23390 given before conditioning or before both conditioning and retrieval decreased freezing at retrieval, whereas SCH23390 given only before retrieval had no effect. In experiment 2, SCH23390 infused into dmPFC before conditioning decreased freezing at retrieval, while infusion of SCH23390 into NAc or VH had no effect.

**Conclusions:**

The results of experiment 1 confirm those of previous studies indicating that D1Rs are required for the acquisition but not retrieval of contextual fear and rule out state dependency as an explanation for these findings. Moreover, the results of experiment 2 provide evidence that dmPFC is also part of the neural circuitry through which D1R signalling regulates contextual fear conditioning.

## Introduction

Dopamine is a neurotransmitter that is important for memory processing and its role in mediating appetitive learning is well established (Dalley and Everitt 2009). However, dopamine transmission is also involved in various aspects of aversive learning and memory (Pezze and Feldon 2004; Brandão et al. 2015). During contextual fear conditioning, a novel context is paired with an aversive unconditioned stimulus (US; e.g., footshock). This leads to an association between the context and US forming and, in turn, a fear response being elicited in the context after conditioning. Dopamine D1-like receptor (D1R) signalling has been linked to this type of aversive learning. Systemic D1R blockade with the selective antagonist SCH23390 impairs contextual fear conditioning, as shown by a decrease in freezing during later retrieval testing (Inoue et al. 2000; Calzavara et al. 2009; Heath et al. 2015). In contrast, SCH23390 given immediately after conditioning or before retrieval has no effect on fear at retrieval (Inoue et al. 2000; Bai et al. 2009; Heath et al. 2015). These findings suggest that D1R antagonism disrupts the acquisition but not the consolidation or retrieval of contextual fear, although it is possible that the effect of SCH23390 on contextual fear conditioning in these previous studies involved state dependency. Memory retrieval can be enhanced when the internal state during retrieval is similar to when the memory was originally acquired (Overton 1964). However, previous reports of SCH23390-induced impairment of the acquisition of contextual fear did not address potential drug effects on state-dependent learning given that retrieval was tested drug-free.

The neural substrates underpinning the effect of SCH23390 on contextual fear conditioning also remain to be fully elucidated. Heath et al. (2015) showed that SCH23390 impairs the acquisition of contextual fear when infused into the dorsal hippocampus (DH) or basolateral amygdala (BLA). These two brain regions are crucial for contextual fear conditioning, with encoding of the contextual representation and context-US association thought to be mediated by DH and BLA, respectively (Anagnostaras et al. 2001). However, D1Rs in other areas may also be involved in regulating contextual fear conditioning. DH and BLA are reciprocally connected indirectly via the ventral hippocampus (VH) (Pitkanen et al. 2000), which is crucial for regulating innate fear but is also involved in spatial and contextual fear learning (Bast et al. 2001; Kjelstrup et al. 2002, 2008; Sutherland et al. 2008; Trent and Menard 2010; Czerniawski et al. 2012; de Hoz and Martin 2014; Zhang et al. 2001, 2014). VH receives dopamine input and expresses D1Rs (Fremeau Jr et al. 1991; Gasbarri et al. 1994), suggesting that D1R signalling in this area might regulate contextual fear conditioning. The hippocampus and BLA project to corticostriatal areas, such as the dorsomedial prefrontal cortex (dmPFC) and the nucleus accumbens (NAc) (McDonald 1991; Thierry et al. 2000), traditionally linked to executive and motivational functions but which also form part of the wider neural circuitry underlying contextual fear conditioning (Haralambous and Westbrook 1999; Levita et al. 2002a; Thomas et al. 2002; Dalley et al. 2004; Cassaday et al. 2005; Liljehom and O’Doherty 2012; Einarsson and Nader 2012; Gilmartin et al. 2013; de Lima et al. 2018). Both dmPFC and NAc receive dopaminergic projections, and D1Rs are expressed in these regions (Oades and Halliday 1987; Fremeau Jr et al. 1991). Moreover, dopamine and D1Rs in these areas have been implicated in various contextual fear processes (Pezze et al. 2001, 2016; Martinez et al. 2008; Albrechet-Souza et al. 2013; Ikegami et al. 2014), although it remains to be established if D1R signalling in dmPFC and NAc is required for contextual fear conditioning.

In this study, we sought to confirm and extend previous findings on the effects of SCH23390 on contextual fear conditioning. In experiment 1, we determined the effects of systemic SCH23390 administration on the acquisition and retrieval of contextual fear, and if any observed drug effects on these processes reflected state dependency. In experiment 2, we examined the effects of infusing SCH23390 locally into the VH, dmPFC, or NAc on contextual fear conditioning. We also assessed the effects of SCH23390 infusion into these areas on behavior in the open-field test to confirm if D1Rs in these areas are involved in mediating the impairing effect of this drug on locomotor activity (Bruhwyler et al. 1991; Heath et al. 2015). This has implications for interpreting any acute drug effects on freezing during conditioning, given that changes in locomotion may affect the expression of freezing.

## Materials and methods

### Animals

Male Lister Hooded rats (Charles River, UK) weighing 280–390 g at the start of the experiment (experiment 1) or before surgery (experiment 2) were used. Rats were group housed in individually ventilated cages and kept on a 24-h light/dark cycle (lights on at 07.00) with ad libitum access to food and water. All behavioral testing occurred during the rats’ light cycle. The principles of laboratory animal care were followed and all experimental procedures were performed with institutional ethical approval and under the UK Animals (Scientific Procedures) Act 1986 (Home Office Project Licence 30/3230).

### Experiment 1

#### Drug injection

SCH23390 hydrochloride (0.1 mg/kg, i.p.; Tocris Bioscience, UK) was dissolved in 0.9% sterile saline. This dose has previously been shown to disrupt contextual fear conditioning (Inoue et al. 2000; Heath et al. 2015). Vehicle-treated controls received injections of 0.9% sterile saline (1 mL/kg, i.p.).

#### Contextual fear conditioning and memory testing

**Fig. 1:**
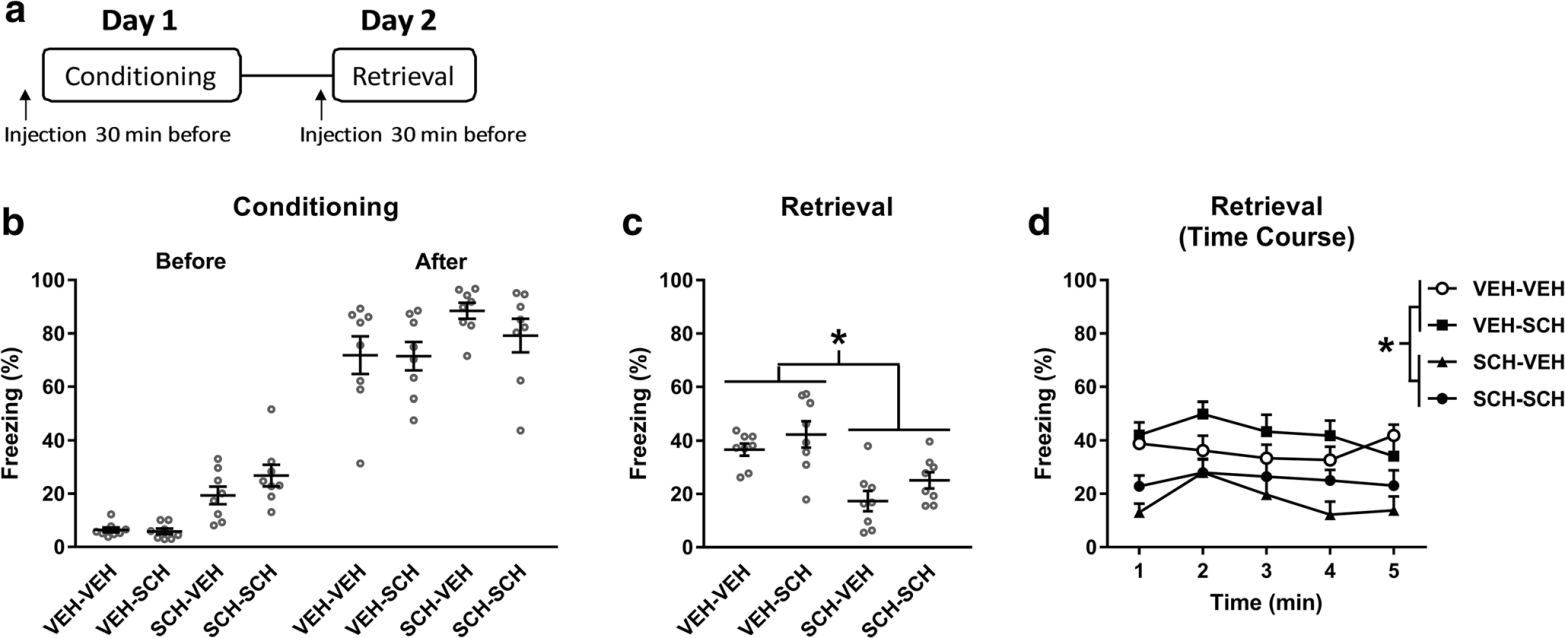
SCH23390 impairment of contextual fear conditioning is not accounted for by state dependency. **a** Schematic representation of the experimental design used. **b** SCH23390 injection before conditioning (SCH-VEH and SCH-SCH) increased freezing before and after shock presentations, compared to vehicle (VEH-VEH and VEH-SCH) (*P* < 0.05). **c** There was no difference in freezing between the VEH-VEH and VEH-SCH groups throughout retrieval, whereas freezing in the SCH-VEH and SCH-SCH groups was decreased, compared to the VEH-VEH and VEH-SCH groups (*P < 0.05). **d** The time course analysis of freezing during retrieval also showed that freezing in the SCH-VEH and SCH-SCH groups was decreased, compared to the VEH-VEH and VEH-SCH groups (*P < 0.05)

The effects of systemic administration of SCH23390 on contextual fear learning and memory retrieval were investigated using a 2-day paradigm (Fig. 1a). The apparatus used has been described in detail elsewhere (Stevenson et al. 2009), and the procedures used were modified from our previous studies (Stevenson 2011; Heath et al. 2015). On day 1, rats were conditioned in a novel context consisting of distinct visual (stripes or spots on two walls of the chambers with the house light on), auditory (60-dB white noise), and olfactory (40% ethanol) cues present during conditioning. The US used was a mild electric shock delivered automatically through the floor bars of the chamber by a computer running MED-PC IV software (Med Associates, VT). Rats were placed in one of the four chambers and after 2 min received four unsignalled shocks (0.5 mA, 0.5 s, 1-min inter-trial interval); rats were removed from the chamber 2 min after the last shock and returned to the home cage. On day 2, rats were returned to their conditioning chamber for 5 min to test retrieval. Rats were randomly allocated to receive an injection of SCH23390 or vehicle 30 min before conditioning and/or retrieval, resulting in the following treatment groups: (1) vehicle given before conditioning and retrieval (VEH-VEH), (2) vehicle given before conditioning and SCH23390 given before retrieval (VEH-SCH), (3) SCH23390 given before conditioning and vehicle given before retrieval (SCH-VEH), and (4) SCH23390 given before conditioning and retrieval (SCH-SCH). Behavior on both days was recorded using a digital camera (Viewpoint, France) positioned above the chamber. The floor bars and waste tray were cleaned with 40% ethanol between each session. Rats were tested at approximately the same time of day (± 1 h) on both days.

#### Data analysis

Freezing was scored automatically using Videotrack Software (Viewpoint). The freezing detection threshold was set to detect the absence of movement apart from that related to respiration. During conditioning, the cumulative duration of freezing during the 2-min intervals before the first and after the last shock was calculated and expressed as a percentage of both 2-min durations. Differences in freezing before and after conditioning between the four groups were analyzed using a two-way analysis of variance (ANOVA), with treatment as the between-subject factor and time (i.e., before and after conditioning) as the within-subject factor. Freezing during the retrieval session was determined as above. Differences in freezing during retrieval testing between the four groups were analyzed in two ways. Freezing throughout the whole session was analyzed using a one-way ANOVA, with treatment as the between-subject factor. Freezing during each 1-min bin of the session was also analyzed using a two-way ANOVA, with treatment as the between-subject factor and time as the within-subject factor. Post hoc analysis was conducted using the Newman-Keuls test where indicated. The data are presented in scatter plots, with the mean ± SEM indicated by horizontal lines and error bars, respectively, or in line graphs as the mean + SEM. The level of significance for all comparisons was set at *P* < 0.05.

### Experiment 2

#### Surgery

Anesthesia was induced with ~ 3% isoflurane in O_2_ and an opioid analgesic (buprenorphine, 0.05 mg/kg, Richter Pharma, Austria) was administered immediately post-induction. Anesthesia was maintained during surgery with 1.5–2.5% isoflurane to ensure complete inhibition of the hindpaw withdrawal reflex. Rats were placed in a stereotaxic frame and the incisor bar was adjusted to maintain the skull horizontal. Rats were implanted bilaterally with guide cannulae (26 gauge, PlasticsOne, VA) fitted with obturators (33 gauge; PlasticsOne) targeting dmPFC (2.7–3.0 mm anterior and 1.2 mm lateral (angled 12° from the midline) to bregma, 2.0–2.3 mm ventral to the brain surface), NAc (1.2 mm anterior and 2.4 mm lateral (angled 6° from the midline) to bregma, 6.5 mm ventral to the brain surface), or VH (5.0 mm posterior and 4.8 mm lateral to bregma, 6.3 mm ventral to the brain surface) (Paxinos and Watson 2007). Cannulae were secured with dental cement to 4–6 screws threaded into the skull. At the end of surgery, a non-steroidal anti-inflammatory analgesic (Metacam, 1 mg/kg, Boehringer Ingelheim, Germany) was administered. Rats were singly housed for 1–2 days post-surgery to allow time to recover without their cagemates being present and potentially interfering with the wound or implant, after which they were group housed as above. Buprenorphine and Metacam were given for 2 days following surgery. Two days after surgery, rats were mildly restrained and the obturators were replaced with clean ones. On days 4 and 6 after surgery, the obturators were loosened and re-tightened. This ensured that the cannulae remained unblocked after surgery and also served to habituate the rats to handling for the local drug infusion procedure (see below). Behavioral testing commenced 6–7 days after surgery.

#### Drug infusion

SCH23390 (2.5 μg) was dissolved in 0.5 μL of 0.9% sterile saline. This dose has previously been shown to impair contextual fear conditioning when infused locally into the BLA (Heath et al. 2015). SCH23390 or vehicle (0.9% sterile saline) was infused bilaterally into dmPFC, NAc, or VH in a volume of 0.5 μL over 1 min using injector cannulae (33 gauge; PlasticsOne) connected to 1 μL Hamilton syringes via polyethylene tubing. The injector cannulae were left in place for 1 min following infusions to allow for drug diffusion and were then removed and replaced with the obturators.

#### Contextual fear conditioning and memory testing

The effects of infusing SCH23390 into dmPFC, NAc, or VH on contextual fear conditioning were investigated using a 2-day testing paradigm (Fig. 3a). Rats were randomly allocated to receive a local infusion of SCH23390 or vehicle 10 min before conditioning on day 1 and retrieval was tested drug-free on day 2. The apparatus and procedures used were the same as above for experiment 1, except that the US duration was extended to 1 s to mitigate any potential deficit in freezing caused by surgery (Zhang et al. 2001; Hart et al. 2009; Heath et al. 2015).

#### Open-field testing

The same rats used in the contextual fear conditioning experiment were used 2–4 days later for open-field testing. The apparatus and testing procedures used have been described in detail elsewhere (Heath et al. 2015). Rats were randomly allocated to receive a local infusion of SCH23390 or vehicle as described above. Open-field testing, which commenced 10 min after drug infusion, lasted for 10 min. Behavior was digitally recorded for subsequent data analysis.

#### Histology

**Fig. 2:**
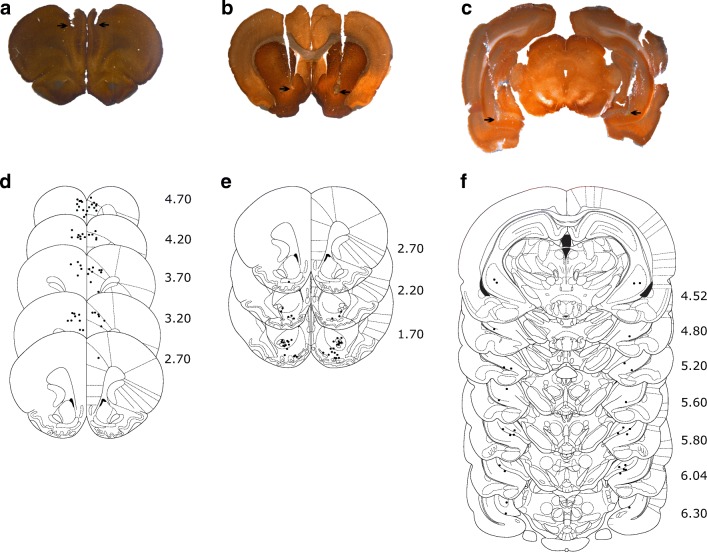
Representative cannulae tip placements in dmPFC (**a**), NAc (**b**), and VH (**c**), as indicated by the arrows. Schematic representation of cannula tip placements in dmPFC (**d**), NAc (**e**), and VH (**f**), with the distance (mm) anterior (**d**, **e**) or posterior (**f**) to bregma indicated to the right

Upon completion of open-field testing, rats were deeply anesthetized with sodium pentobarbital and perfused transcardially with 0.9% saline followed by 4% paraformaldehyde. The brains were removed, post-fixed in 4% paraformaldehyde, and kept at 4 °C until slicing. The sections containing the relevant areas were obtained and stained for acetylcholinesterase. Only data from rats with histologically confirmed cannula placements bilaterally in dmPFC (prelimbic or rostral anterior cingulate cortices), NAc (core or shell), and VH (CA1) were included in the analysis (Fig. 2).

#### Data analysis

Freezing before the first and after the last shock presentations during conditioning was quantified as above for experiment 1. Differences in freezing before and after conditioning between the two groups were analyzed using a two-way ANOVA, with treatment as the between-subject factor and time as the within-subject factor. Freezing during retrieval was quantified as above for experiment 1, and differences in freezing during retrieval testing between the two groups were analyzed in two ways. Freezing throughout the whole session was analyzed using two-tailed unpaired *t* tests. Freezing during each 1-min bin of the session was also analyzed using a two-way ANOVA, with treatment as the between-subject factor and time as the within-subject factor. Separate analyses were conducted for each brain area. Digitally recorded behavior in the open field was analyzed using Ethovision software (Noldus, Netherlands). The percentage time spent in the center of and the total horizontal distance moved in the open field were determined and taken as indices of innate fear and locomotor activity, respectively (Prut and Belzung 2003). Differences in these behavioral measures between the two groups were analyzed separately using two-tailed unpaired *t* tests. Again, separate analyses were conducted for each brain area. Post hoc analysis was conducted using the Newman-Keuls test where indicated. The data are presented in scatter plots, with the mean ± SEM indicated by horizontal lines and error bars, respectively, or in line graphs as the mean + SEM. The level of significance for all comparisons was set at *P* < 0.05.

## Results

### Experiment 1

#### SCH23390 impairment of contextual fear conditioning is not accounted for by state dependency

To determine if disrupted contextual fear conditioning caused by systemic SCH23390 treatment involves state dependency, we examined the effects of SCH23390 given before conditioning and/or retrieval testing (*n* = 8/group). The effects of SCH23390 given before conditioning on freezing before and after shock presentations during conditioning are shown in Fig. 1b. Two-way ANOVA revealed significant main effects of treatment (*F*_(3, 42)_ = 6.28, *P* = 0.0013) and time (*F*_(1, 14)_ = 754.6, *P* < 0.0001) but no treatment *x* time interaction (*F*_(3, 42)_ = 1.20, *P* = 0.32). Post hoc analysis indicated that freezing was significantly increased in the SCH-VEH and SCH-SCH groups, compared to the VEH-VEH and VEH-SCH groups, across both time points (i.e., before and after shock presentations) (*P* < 0.05). This indicates that SCH23390 increased freezing acutely during contextual fear conditioning.

The effects of SCH23390 given before conditioning and/or retrieval on freezing at retrieval are shown in Fig. 1c, d. For freezing throughout the whole retrieval session (Fig. 1c), one-way ANOVA revealed a significant main effect of treatment (*F*_(3, 28)_ = 9.46, *P* = 0.0002). Post hoc analysis found no difference in freezing between the VEH-VEH and VEH-SCH groups (*P* > 0.05), indicating that SCH23390 had no effect on contextual fear retrieval. However, freezing in the SCH-VEH group was significantly decreased, compared to both the VEH-VEH and VEH-SCH groups (*P* < 0.01), suggesting that SCH23390 impaired contextual fear conditioning. Importantly, freezing in the SCH-SCH group was also significantly decreased, compared to both the VEH-VEH and VEH-SCH groups (*P* < 0.05). This was confirmed by the more detailed time course analysis (Fig. 1d). Two-way ANOVA revealed a significant main effect of treatment (*F*_(3, 28)_ = 9.46; *P* = 0.0002) but no main effect of time (*F*_(4, 112)_ = 2.27; *P* = 0.066) or treatment *x* time interaction (*F*_(12, 112)_ = 1.11; *P* = 0.36). Post hoc analysis indicated that, again, while there was no difference between the VEH-VEH and VEH-SCH groups or between the SCH-VEH and SCH-SCH groups (*P* > 0.05), freezing in the SCH-VEH and SCH-SCH groups was significantly decreased, compared to the VEH-VEH and VEH-SCH groups (*P* < 0.05). These results indicate that the effect of SCH23390 on contextual fear conditioning does not reflect drug effects on state-dependent learning but rather that SCH23390 disrupts contextual fear encoding.

### Experiment 2

#### SCH23390 infusion into dmPFC, but not NAc or VH, impairs contextual fear conditioning

**Fig. 3:**
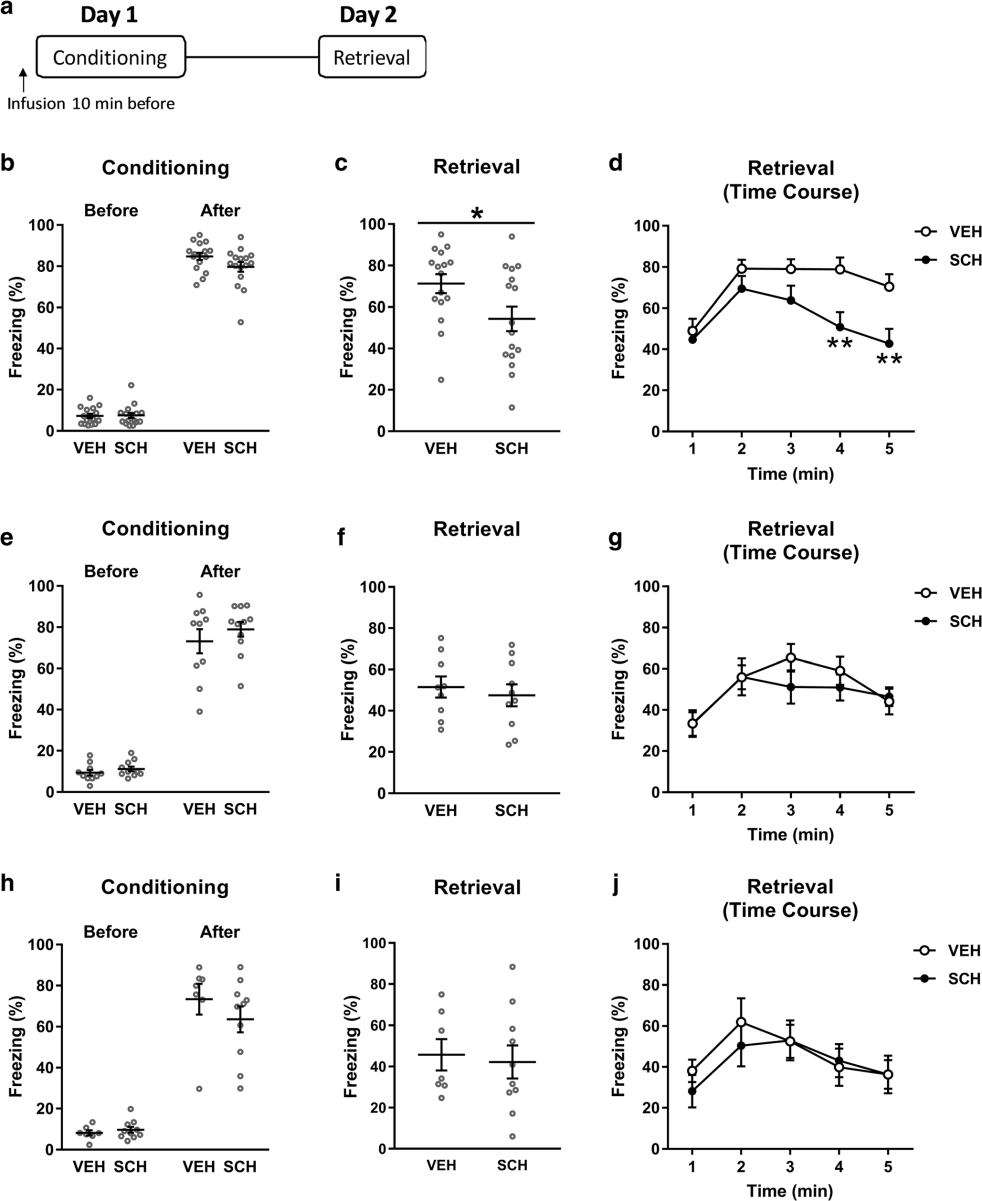
SCH23390 infusion into dmPFC, but not NAc or VH, impairs contextual fear conditioning. **a** Schematic representation of the experimental design used. **b** Infusion of SCH23390 into dmPFC before conditioning had no effect on freezing during conditioning. **c** SCH23390 infused into dmPFC before conditioning decreased freezing during retrieval, compared to vehicle, over the whole session (*P < 0.05). **d** This effect of intra-dmPFC SCH23990 infusion was due to a decrease in freezing in the fourth and fifth minute of the retrieval session (**P < 0.01). SCH23390 infusion into NAc before conditioning had no effect on freezing during conditioning (**e**) or retrieval (**f**, **g**). SCH23390 infused into the VH was also without effect on freezing during conditioning (**h**) or retrieval (**i**, **j**)

SCH23390 was previously shown to impair the acquisition of contextual fear when infused into the DH or BLA (Heath et al. 2015). To determine if D1R modulation of contextual fear conditioning also involves other brain areas implicated in contextual fear processing, we examined the effects of infusing SCH23390 into dmPFC, NAc, or VH on the acquisition of contextual fear. The effect of SCH23390 infusion into dmPFC before conditioning on freezing before and after shock presentations during conditioning is shown in Fig. 3b. Two-way ANOVA revealed a significant main effect of time (*F*_(1, 30)_ = 1493, *P* < 0.0001) but no main effect of treatment (*F*_(1, 30)_ = 3.12, *P* = 0.087) or treatment *x* time interaction (*F*_(1, 30)_ = 1.88, *P* = 0.18). This indicates that SCH23390 (*n* = 16) had no effect on freezing, compared to vehicle (*n* = 16), during conditioning. The effect of intra-dmPFC infusion of SCH23390 before conditioning on freezing during retrieval is shown in Fig. 3c, d. SCH23390 significantly decreased freezing over the entire retrieval session, compared to vehicle (*t*_(30)_ = 2.27, *P* = 0.031), indicating that SCH23390 infusion into dmPFC disrupted contextual fear conditioning (Fig. 3c). The time course analysis showed that this effect of SCH23390 was driven by decreased freezing during later retrieval (Fig. 3d). Two-way ANOVA revealed significant main effects of treatment (*F*_(1, 30)_ = 5.13; *P* = 0.031) and time (*F*_(4, 120)_ = 21.04; *P* < 0.0001), and a significant treatment *x* time interaction (*F*_(4, 120)_ = 4.67; *P* = 0.0015). Post hoc analysis showed that intra-dmPFC SCH23390 infusion significantly decreased freezing during the fourth and fifth minute of the retrieval session (*P* < 0.01).

The effect of infusing SCH23390 into the NAc before conditioning on freezing before and after shock presentations during conditioning is shown in Fig. 3e. Two-way ANOVA revealed a significant main effect of time (*F*_(1, 19)_ = 381.1, *P* < 0.0001) but no main effect of treatment (*F*_(1, 19)_ = 1.11, *P* = 0.30) or treatment *x* time interaction (*F*_(1, 19)_ = 0.36, *P* = 0.56). This indicates that there was no effect of SCH23390 (*n* = 10), compared to vehicle (*n* = 9), on freezing during conditioning. The effect of SCH23390 infusion into the NAc before conditioning on freezing at retrieval is shown in Fig. 3f, g. SCH23390 also had no effect on freezing, compared to vehicle (*t*_(17)_ = 0.54, *P* = 0.60), over the whole retrieval session (Fig. 3f). This was confirmed by the time course analysis (Fig. 3g). Two-way ANOVA revealed a significant main effect of time (*F*_(4, 68)_ = 9.64; *P* < 0.0001) but no main effect of treatment (*F*_(1, 17)_ = 0.29; *P* = 0.60) or treatment *x* time interaction (*F*_(4, 68)_ = 1.08; *P* = 0.38). These results indicate that SCH23390 infusion into the NAc did not affect contextual fear conditioning.

The effect of infusing SCH23390 into the VH before conditioning on freezing before and after shock presentations during conditioning is shown in Fig. 3h. Again, two-way ANOVA revealed a significant main effect of time (*F*_(1, 15)_ = 158.6, *P* < 0.0001) but no main effect of treatment (*F*_(1, 15)_ = 0.64, *P* = 0.44) or treatment *x* time interaction (*F*_(1, 15)_ = 1.44, *P* = 0.25). This indicates that there was no effect of SCH23390 (*n* = 10), compared to vehicle (*n* = 7), on freezing during conditioning. The effect of intra-VH SCH23390 infusion before conditioning on freezing during retrieval is shown in Fig. 3i, j. SCH23390 also had no effect on freezing, compared to vehicle (*t*_(15)_ = 0.31, *P* = 0.76), over the entire retrieval session (Fig. 3i). This was confirmed by the time course analysis (Fig. 3j). The two-way ANOVA revealed a significant main effect of time (*F*_(4, 60)_ = 10.82; *P* < 0.0001) but no main effect of treatment (*F*_(1, 15)_ = 0.093; *P* = 0.76) or treatment *x* time interaction (*F*_(4, 68)_ = 1.18; *P* = 0.33). These results indicate that SCH23390 infusion into the VH also had no effect on contextual fear conditioning.

#### SCH23390 infusion into NAc, but not dmPFC or VH, reduces locomotor activity in the open field

**Fig. 4:**
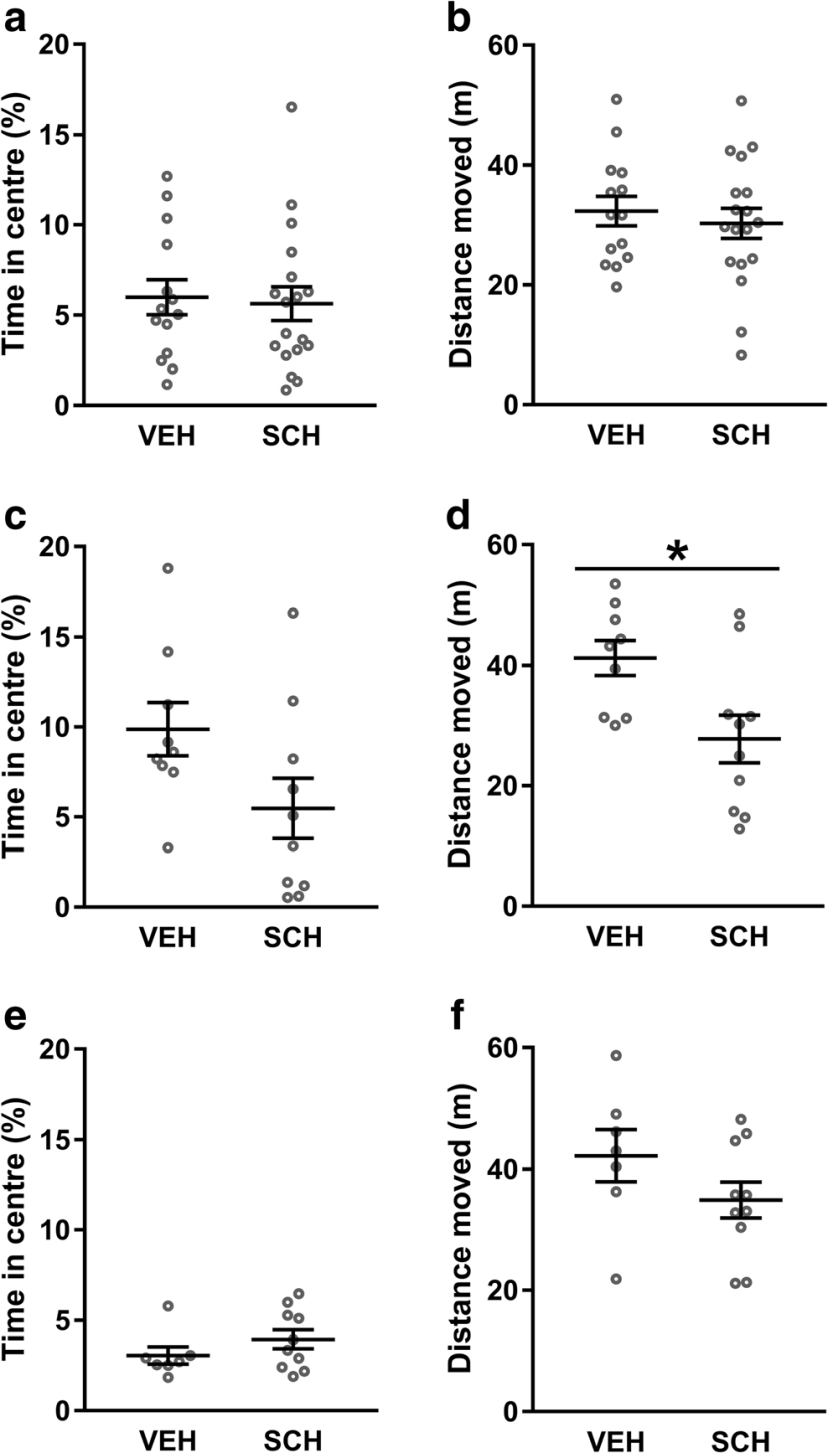
SCH23390 infusion into NAc, but not dmPFC or VH, reduces locomotor activity in the open-field test. SCH23390 infusion into dmPFC had no effect on innate fear, measured as the time spent in the center (**a**), or locomotor activity, measured as the horizontal distance moved (**b**), in the open-field test. Infusing SCH23390 into NAc had no significant effect on innate fear (**c**) but it did decrease locomotor activity (**d**), compared to vehicle (*P < 0.05). SCH23390 infused into VH had no effect on innate fear (**e**) or locomotor activity (**f**)

We also examined the effects of infusing SCH23390 into dmPFC, NAc, or VH on locomotor activity and innate fear during open-field testing to determine if the effects of systemic SCH23390 treatment reported in previous studies involve these areas. The effects of SCH23390 infusion into dmPFC on behavior in the open-field test are shown in Fig. 4a, b. Compared to vehicle (*n* = 14), SCH23390 (*n* = 18) had no effect on the percentage of time spent in the center (*t*_(30)_ = 0.26, *P* = 0.79; Fig. 4a) or the distance moved (*t*_(30)_ = 0.58, *P* = 0.57; Fig. 4b) in the open field. The effects of infusing SCH23390 into the NAc on behavior during open-field testing are shown in Fig. 4c, d. SCH23390 (*n* = 10) had no significant effect on the percentage of time spent in the center (*t*_(17)_ = 1.95, *P* = 0.067; Fig. 4c) but did significantly decrease the distance moved (*t*_(17)_ = 2.68, *P* = 0.016; Fig. 4d) in the open field, compared to vehicle (*n* = 9). The effects of intra-VH infusion of SCH23390 on behavior in the open-field test are shown in Fig. 4e, f. There were no effects of SCH23390 (*n* = 10) on the percentage of time spent in the center (*t*_(15)_ = 1.20, *P* = 0.25, Fig. 4e) or the distance moved (*t*_(15)_ = 1.44, *P* = 0.17; Fig. 4f) during open-field testing, compared to vehicle (*n* = 7). These results indicate that, while SCH23390 does not act in any of these areas to regulate innate fear, the NAc is a site of action for the modulatory effects of this drug on locomotor activity.

## Discussion

This study investigated the role of D1Rs in modulating contextual fear conditioning in two ways. First, we determined if the impairing effect of systemic SCH23390 administration on the acquisition of contextual fear reported previously involves state dependency. In experiment 1, we confirmed previous results indicating that SCH23390 treatment disrupts contextual fear conditioning but not its retrieval. We also extended these findings by showing that its effect on the acquisition of contextual fear was not attributable to a state-dependent effect of this drug, given that SCH23390 administered before both learning and memory testing still resulted in impaired contextual fear conditioning. Second, we further characterized the neural substrates involved in mediating D1R regulation of contextual fear conditioning by examining the effect of infusing SCH23390 into VH, dmPFC, or NAc on the acquisition of contextual fear. In experiment 2, we found that SCH23390 infusion into dmPFC, but not VH or NAc, disrupted the acquisition of contextual fear, indicating that D1R signalling in dmPFC is involved in regulating contextual fear conditioning. We also showed that SCH23390 infused into the NAc, but not VH or dmPFC, reduced locomotor activity in the open-field test, confirming previous findings indicating that D1Rs in NAc play a role in modulating locomotion.

Our results showing that systemic SCH23390 treatment impaired the acquisition but not retrieval of contextual fear confirm previous findings (Inoue et al. 2000; Bai et al. 2009; Calzavara et al. 2009; Heath et al. 2015). SCH23390 enhanced freezing during conditioning, whereas it may have been expected that impaired acquisition would be associated with decreased freezing after shock presentations during conditioning. This finding is difficult to interpret though, given that SCH23390 also reduces locomotor activity acutely (Bruhwyler et al. 1991; Heath et al. 2015), which might resemble enhanced freezing. However, during retrieval, SCH23390 had no significant effect on freezing acutely. One possible explanation for this discrepancy is that novelty, which is associated with a dopamine-dependent increase in locomotion (Blanchard et al. 2009), was a factor during conditioning but not at retrieval. It is worth noting that, while systemic SCH23390 administration did not affect contextual fear retrieval, local infusions of this drug have been shown to disrupt the retrieval of different types of fear memory (Lamont and Kokkinidis 1998; Nader and LeDoux 1999), including contextual fear (Albrechet-Souza et al. 2013), possibly indicating opposing effects in different brain areas resulting in no net effect when SCH23390 is given systemically.

Our results also provide evidence that the disruptive effect of SCH23390 on the acquisition of contextual fear does not reflect state dependency. Previous studies did not examine the effects of SCH23390 on contextual fear conditioning with drug given before both conditioning and retrieval; therefore, the reported effects of SCH23390 may have been due to a mismatch between the drug-induced internal state during conditioning and retrieval. If SCH23390 given before conditioning and retrieval had no effect on freezing at retrieval, then this would support the interpretation that SCH23390-induced impairment of contextual fear conditioning reflected drug effects on state-dependent learning. Alternatively, if SCH23390 given before conditioning and retrieval results in reduced freezing at retrieval, as we found in the present study, then this provides evidence that the impairing effect of SCH23390 on contextual fear conditioning cannot be accounted for by state dependency. This finding is congruent with previous studies showing that state-dependent drug effects did not explain the disruptive effects of local infusions of dopamine receptor antagonists on spatial learning or cued fear retrieval (Pezze et al. 2003; O’Carroll et al. 2006).

In terms of the brain areas involved in mediating its effect, we found that infusing SCH23390 into dmPFC impaired contextual fear conditioning. This indicates that D1Rs in this area contribute to the acquisition of contextual fear, which is in general agreement with recent studies showing that D1R activation in dmPFC facilitates the encoding of fear memory involving weaker contextual cues (Pezze et al. 2016; Castillo Díaz et al. 2017). How D1R signalling in dmPFC modulates contextual fear conditioning remains unclear, but there are several possibilities. D1Rs in this area are crucial for attentional processing (Dalley et al. 2004), suggesting that their antagonism may impair the acquisition of contextual fear by disrupting attention to contextual cues during conditioning. D1R signalling in dmPFC may also modulate functional interactions between dmPFC and hippocampus. VH stimulation increases dopamine release in dmPFC (Peleg-Raibstein et al. 2005). Moreover, D1R signalling modulates synchronized neural activity and long-term potentiation in the hippocampo-prefrontal pathway (Jay et al. 2004; Werlen and Jones 2015). This suggests that local blockade of D1Rs may interfere with synaptic plasticity in dmPFC related to contextual encoding conveyed by the hippocampus. The dmPFC is also important for mediating the affective component of pain processing, which is thought to involve input from BLA (Neugebauer 2015). Although systemic SCH23390 administration has no effect on shock sensitivity (Inoue et al. 2000; Heath et al. 2015), dopamine in dmPFC does regulate pain processing, and synaptic plasticity in the amygdala-prefrontal pathway is modulated by dopamine (Lopez-Avila et al. 2004; Onozawa et al. 2011). Therefore, blocking D1Rs in this area might also disrupt affective aspects of pain processing related to the US conveyed by BLA. Further research is needed to determine the precise role of dmPFC D1R signalling in contextual fear conditioning.

In contrast to dmPFC, SCH23390 infusions into the NAc had no effect on the acquisition of contextual fear, although they did have a clear behavioral effect in the open-field test (see below). Both the hippocampus and BLA project to NAc, making this area well-placed for integrating contextual and associative aspects of contextual fear conditioning (McDonald 1991; Thierry et al. 2000). Excitotoxic lesions or temporary inactivation of the NAc impairs the acquisition of contextual fear (Haralambous and Westbrook 1999; Levita et al. 2002a). Dopamine and D1R signalling in this area have also been implicated in contextual fear conditioning and its retrieval in some (Saulskaya and Marsden 1995; Pezze et al. 2001; Martinez et al. 2008; Albrechet-Souza et al. 2013; Ikegami et al. 2014), but not all (Levita et al. 2002b), previous studies. Differences in methodological procedures (e.g., foreground vs background contextual cues) and anatomical specificity (e.g., NAc core vs shell subregions and rostral vs caudal NAc) between the studies may account for some of these apparent discrepancies (Levita et al. 2002b). Previous studies have suggested that the caudal NAc core and its dopamine innervation are involved in contextual fear conditioning (Levita et al. 2002a, b). Our infusion sites ended up being located more rostrally, but they showed considerable anatomical heterogeneity in terms of their spread across both NAc core and shell. However, it might be expected that diffusion of drug occurred to some extent between NAc shell and the overlying core. In support of this idea, Haralambous and Westbrook (1999) found that neuronal inactivation with infusions of the sodium channel inhibitor bupivicaine into NAc had a similar disruptive effect on contextual fear conditioning as lesions to the caudal NAc core; however, their infusions were spread across the rostrocaudal extent of both NAc core and shell, albeit using a greater infusion volume than we used here. Nevertheless, it is possible that we may have observed an effect of SCH23390 in the present study using drug infusions with more anatomical selectively.

Infusing SCH23390 into the VH was also without effect on the acquisition of contextual fear. This area receives input from the DH and projects to the BLA (Pitkanen et al. 2000), thereby providing a link between brain regions that are pivotal for contextual fear conditioning. Lesions, temporary inactivation, and NMDA receptor antagonism in VH impair this form of aversive learning (Bast et al. 2001; Sutherland et al. 2008; Czerniawski et al. 2012; Zhang et al. 2001, 2014). Taken together with our results, these findings indicate that neuronal activity and synaptic plasticity in this area are necessary for contextual fear conditioning but that local D1R signalling may not be required for this process.

Infusing SCH23390 into dmPFC did not affect the amount of time spent in the center of the open field, indicating that D1R signalling in this area may play different roles in regulating innate fear and contextual fear learning. This lack of effect is consistent with the results of previous studies showing no effect of dmPFC inactivation on innate fear (Corcoran and Quirk 2007; Stevenson 2011). We also found that SCH23390 infused into dmPFC had no effect on locomotor activity, which has also been shown previously (Barros et al. 2001; Shah et al. 2004; Hall et al. 2009). Similarly, SCH23390 infusion into VH had no effect on innate fear or locomotion during open-field testing, confirming previous findings (Giménez-Llort et al. 2002; Andrzejewski et al. 2006; Zarrindast et al. 2010). Lesions or inactivation of VH reduces innate fear in the elevated plus maze (Kjelstrup et al. 2002; Trent and Menard 2010), but our results suggest that D1Rs in this area are not involved in regulating innate fear. In the NAc, we found that SCH23390 reduced locomotion but had no significant effect on innate fear in the open-field test. Again, our results agree with previous studies showing D1R signalling in this area modulates locomotor activity but not innate fear (Dreher and Jackson 1989; Ahmadi et al. 2013).

In conclusion, our results confirm previous findings showing that SCH23390 impairs the acquisition of contextual fear and extends them by showing that this effect is not due to this drug causing effects on state-dependent learning. Our results also provide evidence that D1R regulation of contextual fear conditioning is mediated at least in part by dmPFC, adding to other brain areas (i.e., DH, BLA, and lateral habenula) recently shown to be involved in this process (Heath et al. 2015; Chan et al. 2017). We found that SCH23390 had a greater impairing effect on contextual fear conditioning when given systemically than when it was infused locally into dmPFC, which is perhaps not surprising if other brain regions are also involved in mediating its effect. Moreover, the acute effect of SCH23390 on freezing during conditioning when given systemically was not observed with local infusions into dmPFC, NAc, or VH. This suggests that, again, more than one of these brain areas might be required, or that other brain areas mediate this effect of SCH23390. More generally, this study contributes to a growing body of research demonstrating that dopamine is important for regulating contextual fear processing (Pezze and Feldon 2004; Brandão et al. 2015). Future studies investigating D1R modulation of functional interactions and synaptic plasticity in the DH-BLA-dmPFC network will help to further elucidate the neurochemical and neural circuit basis of contextual fear conditioning.
